# Thrombosis in COVID-19 infection: Role of platelet activation-mediated immunity

**DOI:** 10.1186/s12959-021-00311-9

**Published:** 2021-08-23

**Authors:** Mahin Behzadi Fard, Samaneh Behzadi Fard, Shahin Ramazi, Amir Atashi, Zahra Eslamifar

**Affiliations:** 1 Dezful university of medical sciences, Dezful, Iran; 2 Kerman university of medical sciences, Kerman, Iran; 3 Department of biophysics, faculty of biological sciences, Tarbiat Modares University, Tehran, Iran; 4 Stem cell and tissue engineering research center, Shahroud university of medical sciences, Shahroud, Iran

**Keywords:** SARS-CoV-2, COVID-19, Thrombosis, TLRs, Platelet activation, PAMPs, Proinflammatory cytokines

## Abstract

**Background:**

Thrombosis plays an important role in the Coronavrus Disease 2019 (COVID-19) infection-related complications such as acute respiratory distress syndrome and myocardial infarction. Multiple factors such as oxygen demand injuries, endothelial cells injury related to infection, and plaque formation.

**Main body:**

Platelets obtained from the patients may have severe acute respiratory syndrome coronavirus 2 (SARS-CoV-2) RNA, showing that the increased activation potential recommends platelet can be hyper-activated in severely ill SARS-CoV-2 cases. Platelets contain multiple receptors that interact with specific ligands. Pathogen’s receptors such as Toll-like receptors (TLRs), NOD-like receptor, C-type lectin receptor family, glycoprotein (GP) such as GPαIIbβ3 and GPIbα which allow pathogens to interact with platelets. Platelet TLRs and NOD2 are involved in platelet activation and thrombosis. Accordingly, TLRs are critical receptors that could recognize various endogenous damage-associated molecular patterns and exogenous pathogen-associated molecular patterns (PAMPs). TLRs are considered as important components in the activation of innate immunity response against pathogenic and non-pathogenic components like damaged tissues. TLRs-1,-2,-4,-6,-7 expression on or within platelets has been reported previously. Various PAMPs were indicated to be capable of binding to platelet-TLRs and inducing both the activation and promotion of downstream proinflammatory signaling cascade.

**Conclusion:**

It is possible that the increased TLRs expression and TLR-mediated platelets activation during COVID-19 may enhance vascular and coronary thrombosis. It may be hypothesized using TLRs antagonist and monoclonal antibody against P-selectin, as the marker of leukocyte recruitment and platelet activation, besides viral therapy provide therapeutic advances in fighting against the thrombosis related complications in COVID-19.

## Introduction

Platelets are key cells in thrombosis, as a physiological process in which vessel damage consequently results in clot formation. However, in pathological situation, these may lead to vessel occlusion, ischemia, and tissue damages [1]. Moreover, endothelial cells injury leads to subendothelial exposure, platelet aggregation, and clot formation [2]. Viruses could attach to platelet-plasma or endosomal membrane surface receptors and then active platelets via specific, signaling cascades [3]. Engagement of surface Fc receptors that bind to immunoglobulin-coated virions could also activate platelets [4]. Platelets express pattern recognition receptors (PRRs), including TLR, Nod-like receptor, and C-type lectin receptors that has a critical role in recognition of damage-associated molecular patterns (DAMPs) as well as exogenous pathogen-associated molecular patterns (PAMPs), which is referred as virus associated molecular patterns (VAMPs) in cases of viruses [5–7]. TLRs are critical molecules in the initiation of innate and adaptive immune responses, and may be expressed either on cell surfaces (TLRs-1,-2,-4,-5,-6,-10) or in the endosome compartment (TLRs-3,-7,-8,-9) [8–11]. These receptors attachment to DAMP and PAMP can active intracellular pathways such as production of proinflammatory cytokines [12–15]. Platelets endocytose virions, after attachment of TLRs to their released lysosomal ligands i.e., ssRNA, dsRNA, CpG DNA downstream signaling lead to platelet activation and granule release, expose P-selectin, and finally form platelet leukocyte aggregates (PLAs) [16].

It is likely that thrombosis due to the activation of the innate immune system through TLRs could induce some subsequent vascular occlusive events [17, 18]. Local release of DAMPs during acute myocardial infarction (AMI) is known as an event that triggers proinflammatory TLR activation, which can subsequently aggravate myocardial injury [19, 20]. Notably, various PAMPs are capable of inducing platelet activation [21]. Treatment with pam3CSK4, which is a pharmaceutical agonist of TLR1/TLR2 ligand, could directly induce platelets activation; platelet’s adhesion, aggregation, degranulation, and interaction with leukocytes [12]. Human platelets express TLRs-1,-2,-4,-6 as well as 7 receptors [22–24]. As well, a recent study showed SARS-CoV-2 spike protein could interact with TLRs, especially TLR-4 [25]. It is possible that alternative platelet activation’s pathways may promote recurrent thrombosis in COVID-19 patients.

By considering the presence of some TLRs and NOD2 in platelets, these molecules may play roles in platelet activation and thrombosis in the onset of myocardial infarctions during SARS-CoV-2 infection.

## Platelet activation and P selectin

P-selectin (CD62P) (formerly known as PADGEM and GMP140), is an integral protein that acts as a cell adhesion molecule on the surfaces of the activated endothelial cells as well as the activated platelets to bind to neutrophil and monocytes [26–28]. The soluble form of P-selectin lacks the transmembrane domain that appears to be produced from alternative splicing of pre mRNA [29]. Of note, the primary ligand for P-selectin is P-selectin glycoprotein ligand-1 (PSGL1), which is expressed on almost all leukocytes. P-selectin leads to leukocyte rolling and then acts as the first agent for the leukocyte recruitment [30]. P-selectin is mostly synthesized in endothelial cells and megakaryocyte and then stored in weibel-palade bodies and α-granules, respectively [31]. Moreover, it plays an essential role in the initial recruitment of leukocytes to the site of injury during inflammation. During this process, inflammatory mediators such as interleukin-4 (IL-4), tumor necrosis factor-α (TNF-α), and LPS result in the P-selectin secretion from endothelial cells. Although LPS and TNF-a increase both mRNA and protein levels in murine models, they cannot affect mRNA expression in human endothelial cells, while IL-4 increases P-selectin mRNA in both of them [32–34]. It was shown that the P-selectin overexpression in endothelial cells leads to leukocyte rolling via PSGL-1 and acts as the first agent for the leukocyte recruitment to inflammatory sites [35]. Additionally, P-selectin activates monocyte to produce tissue factor (TF), which is the main activator of extrinsic coagulation cascade [36]. It is noteworthy that the P-selectin-mediated leukocyte recruitment into the lungs during acute respiratory distress syndrome (ARDS) and infusion of anti P-selectin (monoclonal antibody) reduce the severity of ARDS [37]. Soluble P-selectin is parallel to platelet’s activation and thrombosis elevated in ARDS cases compared to control and also in non-survivors compared to survivors [38, 39].

Due to the high prevalence rate of thrombotic complications among COVID-19 patients, a possible role has been suggested for P-selectin in activating intravascular coagulation [39, 40].

## Platelet receptors activation

Platelets included a cytoskeleton and dense tubular system, few mitochondria, storage granules; glycogen, δ and α granules and peroxisomes. The α-granules contain proteins for the platelets hemostatic functions, such as, thrombospondin, platelet factor-4, von willebrand factor (VWF), fibrinogen, P-selectin, CD40 ligand (CD154), β-thromboglobulin, platelet derived growth factor (PDGF), FV, GP IIb/IIIa, δ granules contain nucleotides (ADP and ATP), serotonin, histamine, pyrophosphate, and calcium. Upon platelet activation, granules contents are transfer to platelet membrane or release to extracellular space to further promote platelet adhesion and activation [41].

**Fig. 1:**
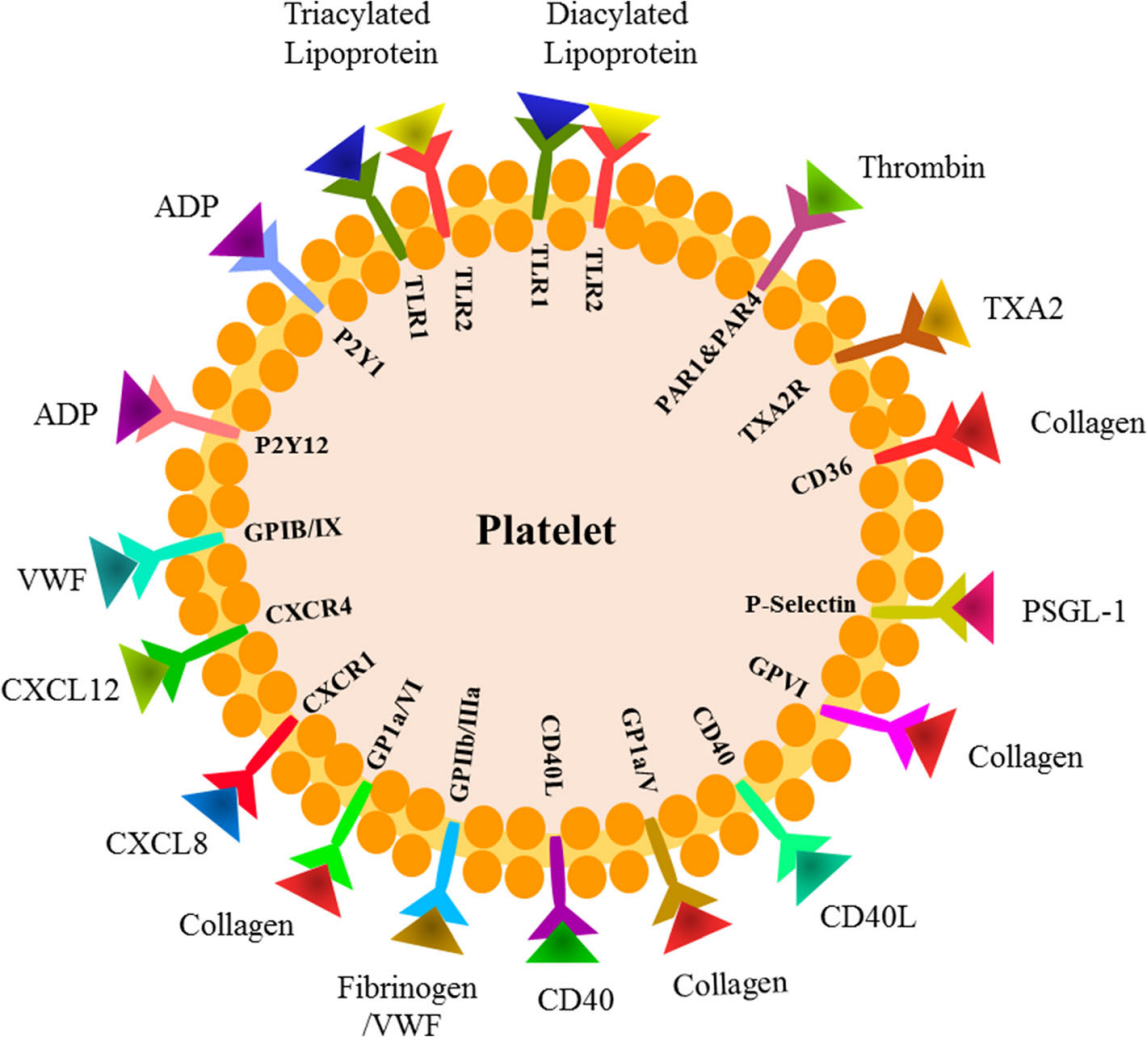
**Different predominant platelet receptors and their physiological role.** PAR1/PAR4; Adhesion, spreading, and secretion, GPIb-IX-V complex;Initiation of platelet recruitment, TXA2R;Platelet aggregation, TLR; Inflammatory response, P2Y1 and P2Y12; Amplification of aggregation, GPIIb/IIIa; Platelet aggregation, CD36;Aggregation, stabilization of aggregates, P-selectin; Clot formation with leukocytes, GPVI; Platelet aggregation, P-selectin interacting with PSGL-1 on leukocyte, CXCR4; Platelet activation (P-selectin expression, adhesion [41]

A wide variety of mobile transmembrane receptors covers the platelet membrane. Many of these receptors are expressed by other cell types, but some are only expressed on platelets. It is well known that the major platelet receptors have a prominent role in the hemostatic function of platelets, allowing platelets have specific interactions and functional responses with vascular adhesive proteins. The platelet receptors including thrombin receptors (PAR-1 and PAR-4), ADP receptors (P2Y_1_ and P2Y_12_), Chemokine receptors (CXCR1 and CXCR4), TxA_2_ receptor, VWF receptor (GPIIb/IIIa), integrins (α_IIb_β_3_, α_2_β_1_, α_5_β_1_, α_6_β_1_, α_V_β_3_), Glycoprotein (GP) Ib/IX/V, Toll-like receptors, proteins belonging to the immunoglobulin superfamily (GP VI, FcγRIIA), P-selectin, CD63, CD36, P-selectin ligand 1, TNF receptor type [3, 6, 9, 42]. (Fig. 1).

## Platelet receptors and viral infection

**Fig. 2:**
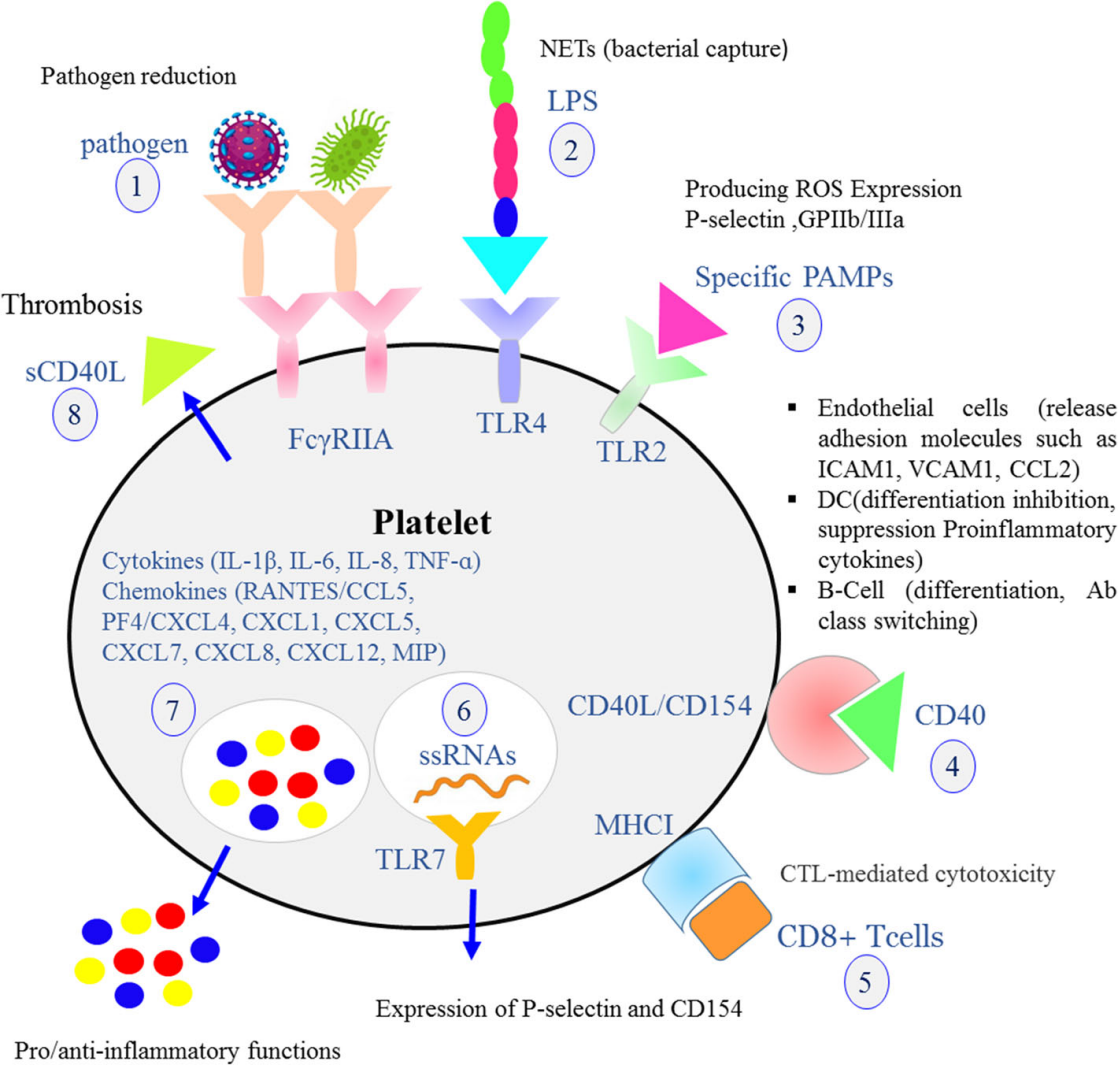
**Platelet immune receptors and functions.** Schematic representation of the crucial roles of platelets in inflammatory processes. 1) The binding of IgG-pathogen complex to IgG receptor (FcγRIIA) leads to pathogen engulfment and reduction. 2,3) Platelets can express TLRs that bind to bacterial LPS and specific PAMPs. 4) Interaction of platelet CD40L with different immune cells has an important role in leukocyte recruitment and activation. 5) Platelet MHC class I molecules are located in intracellular space but upon activation these molecules expressed on cell membrane and can activate antigen-specific CD8+ T cells. 6) Activation of TLR7 that distinguish ssRNAs leads to P-selectin as a platelet activation marker and CD154 receptor. 7) Platelets contain many proinflammatory and anti-inflammatory cytokines and chemokines and be can released from these cells to the extracellular space. 8) Soluble form of CD40L (sCD40L) enhances platelet activation, aggregation, and platelet-leukocyte attachment [3, 6, 42]

Platelets contain multiple receptors that interact with specific ligands. Pathogen’s receptors such as TLRs, NOD like receptors, C-type lectin receptor family, FcγRIIA, glycoprotein (GP) αIIbβ3, GPIbα which allow pathogens to interact with platelets [3, 23, 42, 43]. Innate immune receptors participate in platelet-leukocyte interactions. Pathogens or their products directly or indirectly induce platelet activation. The complex of IgG-pathogen binds to IgG receptor FcγRIIA then leads to pathogen engulfment and reduction. Platelet CD40L expression allows them to interact with different immune cells. In addition, CD40L may be cleaved into a soluble form (sCD40L) that enhances platelet activation, aggregation, and platelet-leukocyte attachment. Platelets can carry and eliminate pathogens, and via the expression of TLRs they can bind bacterial LPS and activate neutrophils, inducing NETs formation. Intact platelet MHC class I molecules are located intracellular but upon activation are expressed and can activate antigen-specific CD8+ T cells. In contrast, the MHC class I molecules on the surface of resting platelets are denatured and lead to CD8+ T cell inhibition. Platelets contain many proinflammatory and anti-inflammatory cytokines and chemokines and, upon activation, can release them to the extracellular space. The culmination of these events makes platelets a main immunomodulatory host (Fig. 2) [6].

Moreover, platelet’s activation during the process of viral exposure by hepatitis virus, adenovirus, Dengue, and HIV-1 was reviewed in a study by Flaujac et al. [44]. Despite the fact that different viruses could activate platelets, its occurrence remained less clear so far. Platelet’s activation after viral exposure can be summarized in the following 3 categories: 1:Viruses may directly bind to surface proteins and then activate platelets via downstream specific signaling cascades [3], 2: Immunoglobulin-coated virions could activate platelets via engagement of surface Fc receptors [4], and 3: TLRs play a role in some viruses (i.e., encephalomyocarditis virus) [45].

## TLRs and platelet activation

Viral ss-RNA of SARS-COV-2 is sensed by TLR-3, TLR-7, and TLR-8 proteins [46]. Previous studies have shown the internalization and clearance of ssRNA viruses such as influenza and HIV by platelets [13, 22, 47]. TLR7 is expressed in platelets at both the protein [48] and mRNA level [49, 50]. Platelets can also internalize pathogens (i.e., bacteria and viruses) when an interaction with endosomal TLRs (e.g., TLR-7 and TLR-9) occurs [12, 13, 51]. Upon viral infection, platelets are activated through TLR7 that change their phenotype and induce the formation of platelet-neutrophil aggregates. After the lysosomal degradation of the internalized viral particles, ssRNA genome attaches to endosomal TLR-7. Virus, via ssRNA may mediate platelets’ activation through TLR-7, leading to degranulation, change of phenotype and aggregates with neutrophils. This mechanism is likely to participate in antiviral immunity since TLR-7-depleted mice had an increase in mortality [16, 45, 52]. The activation of TLR-7 pathway leads to platelet’s degranulation of P-selectin, which consequently results in platelet’s activation as well as overexpression of CD40 ligand CD40L/CD154. These aggregates ultimately lead to platelet-neutrophil aggregates and inflammation and thrombocytopenia without promotion of thrombosis [45]. Platelets stimulate with TLR-3 agonists that lead to α-granule-stored factors translocation (P-selectin and CD40L) to cell surface and induce procoagulant responses to traditional agonists such as thrombin [53, 54]. As TLR-7 recognizes viral ss-RNA, it may be important in platelet’s activation related to SARS-CoV-2 infection. Platelet express TLR-4 that recognize PAMPs and DAMPs ligands [55]. DAMPs like histones, high mobility group box 1 (HMGB1) and heat shock proteins (HSPs) that released during host tissue injury or viral infection can bound to neutrophil extracellular traps, (NETs), trigger both prothrombotic and procoagulant platelet-mediated responses, partly by interacting with TLR4 [56–59].

It is possible that TLRs-mediated platelets activation in COVID-19 patients subsequently exaggerates both vascular and coronary thrombosis and it may also be related to myocardial injury [23, 24].

## Role of platelets in COVID-19 infection

Platelets maintain the integrity of the alveolar capillaries in normal situation, but in pathologic situation, they may contribute into causing lung injury [60]. In addition, platelet-endothelial interactions and platelet-leukocyte aggregation contribute into the pathogenesis of acute lung injury [61–63]. Moreover, in viral infections, thrombocytopenia, interactions with leukocytes, and platelet’s secretion may lead to the protective or injurious immune effects [64].

In patients with COVID-19, thrombocytopenia rate is estimated at about 5–41.7%, and typically it has a mild form (100–150 × 10^9^/L) [65–68]. Additionally, severe thrombocytopenia, which may have an immune mediated source, is rare [69]. It was found that the severe and non-survivors patients have a lower platelet count compared to non-severe and the survivors, respectively [70, 71]. Accordingly, low platelet count in these patients may be due to platelet consumption and associated with the increased risk of mortality; however, it has not been determined as a predictor factor for this disease’s mortality [65, 71]. The patients with a temporal tendency to the decreased platelet count may experience a worsening thrombotic complication and lower nadir platelet counts are related to the increased mortality rate [69, 72].

In viral infections, platelet’s activation may occur either by viral immune complexes or by host inflammatory responses, and the activated platelets are more cleared from circulation by the reticuloendothelial system macrophages [73]. Expression of ACE2, which is the direct receptor of SARS-CoV-2 spike protein on platelets, as well as the induction of platelet’s activation by anti-spike monoclonal antibody were recently reported [74]. A recent report has been shown that circulating platelets obtained from COVID-19 patients had a higher level of surface membranes of P-selectin expression compared to normal controls. Additionally, platelet’s aggregation was greater in patients responding to lower concentrations of platelet agonists [75].

## Toll-like receptors, structures, functions, as well as its specific ligand and main role in thrombosis

TLRs are type I transmembrane glycoprotein (GP) receptors consisting of (i) 20–27 extracellular leucine-rich repeat (LRR) domains used for the recognition of PAMP/DAMP or VAMP, (ii) a transmembrane domain, and (iii) a cytosolic Toll/interleukin (IL)-1 receptor (TIR) domain used for the activation of downstream cell signaling pathways [76]. The extracellular domains of TLR contain glycan moieties serving as binding sites for ligands. Moreover, TLRs are classified according to their ligands and cellular localizations. Immune cells (including dendritic cells, macrophages, NK cells, T cells, and B cells) as well as non-immune cells (including epithelial and endothelial cells, and fibroblasts) express these receptors [77]. Notably, TLR-1, − 2, − 4, − 5, − 6, and 10 are primarily located at the cell surface, whereas TLR3, 7, 8, and 9 are present on the membranes of surrounding intracellular vesicles, including endosomes, lysosomes, and the endoplasmic reticulum that could recognize pathogenic nucleic acids [8, 78].

**Fig. 3:**
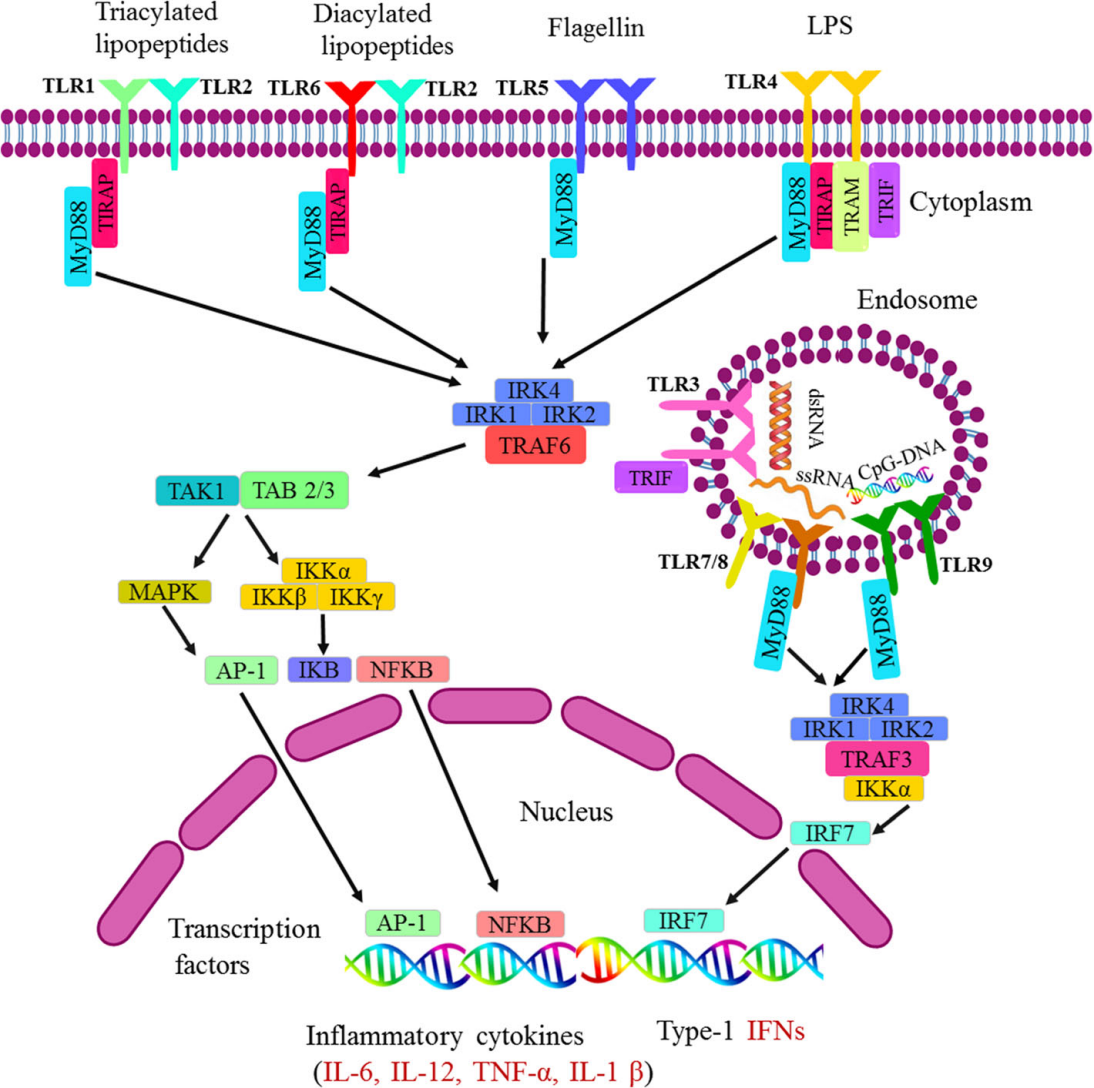
Toll-like receptor (TLR) signaling pathways and involved effector molecules. TLR 1, 2, 4, 5, and 6 are in homodimer or heterodimer forms are located on the cell surfaces, when stimulated by PAMPs, MyD88 interacts with IRAK-4 MyD88-IRAK-4 complex, recruits IRAK-1 and IRAK-2, resulting in the phosphorylation of IRAKs. Phosphorylated IRAKs leave MyD88 after and interact with TRAF6. TRAF6 induces the activation of TAK-1 and TAB2/3 then activation of IκB and MAPK occurs. The activation of IκB and MAPK result in the subsequent translocation of AP1 and NFkB to nucleus. TLR3, 7, 8, and 9 are located on the endosomal membrane. Stimulation of endosomal TLRs leads to the recruitment of MyD88, IRAK4, IRAK1, and TRAF6 and the translocation of IRF7 to nucleusTLR3 and part of TLR4 use TRIF as their adaptor. The interaction of TRIF with RIP1 leads to RIP1 polyubiquitination and their combination with TAB2 and TAB3, result in the translocation of NF-κB and AP-1 nucleus. The nuclear translocation of transcription factors including the NFκB in early and late stages (all TLRs), AP-1 (all except TLR 3), the IRF-3 (TLR3 and TLR4) and IRF-7 (TLR7/8/9) occur. These pathways lead to inflammatory cytokine synthesis, secretion in the case of platelets and the production type 1 IFNs [84, 85]

Furthermore, TLR can be sub-classified based on sequence analysis and three-dimensional structures into three-domain (TLR-1, − 2, − 4, − 6, − 10) and single-domain (TLR-3, − 5, − 7, − 8, − 9) TLRs [76]. In this regard, the single-domain and three-domain TLRs interact with hydrophilic ligands like nucleic acids, and lipid-containing molecules such as LPS and lipoproteins respectively [79]. Platelet’s activation can up regulate TLR-2, TLR-4, and TLR-9 expressions in these cells [23, 80, 81]. As well, in mice, TLR2 ligands can alter megakaryocyte TLRs expression [82]. Platelets express some functional chemokine receptors such as CCR1, 3, 4, and CXCR4, which are involved in infection, hemostasis, inflammation, and even in the development of atherosclerosis (Fig. 3) [42].

A recent study revealed that SARS-CoV-2 mRNAs encoding NSP10, E-protein, NSP8, and S2, have strong binding affinities toward intracellular TLR3, TLR7, TLR8, and TLR9, respectively [83]. Additionally, it was shown that SARS-CoV-2 spike protein could interact with TLR4, and TLR-4 activation plays a major role in inflammatory response in COVID-19 infection.

## NOD2 -related platelet activation and thrombosis

Among the main families of pattern recognition receptors, Toll-like receptors and nucleotide-binding oligomerization domain (NOD)–like receptors are the critical receptors in innate immunity response. NODs are cytoplasmic receptors. NOD1 and NOD2 are the two important NODs, NOD1 and NOD2 contains 1 and 2 caspase recruitment domain respectively [86]. NOD2 is mainly expressed in monocytes, macrophages, dendritic cells, intestinal epithelial cells, and paneth cells whereas NOD1 has a wide distribution. NODs have a major role in innate immune response against infections. In bacterial and viral infections NOD2 through the activation of NF-κB, MAPK and caspase-1 pathways, lead to increase expression of proinflammatory cytokines, including IL-1β, tumor necrosis factor-alpha (TNFα), IL-6, IL-12p40, CC-chemokine ligand 2, IL-8, CXC-chemokine ligand 2 and various antimicrobial agents such as defensins. The NOD2 sensor promotes intestinal pathogen eradication via the chemokine CCL2-dependent recruitment of inflammatory monocytes [87]. NOD2 receptor activation induces platelet production of IL-1β as proinflammatory cytokines [5]. NOD2 as a cytoplasmic viral PRR trigger the activation of interferon-regulatory factor 3 (IRF3) and production of interferon-β (IFN-β). After recognition of a viral ssRNA genome, NOD2 used the adaptor protein mitochondrial antiviral signaling (MAVS) to activate IRF3 and innate immune antiviral response [91]. Similar functions of NOD2 are observed in response to influenza A and parainfluenza viruses [88, 89]. Platelets express NOD2 that potentiates platelet activation and enhances in vivo thrombosis [90]. The crucial role of platelets in thrombosis, hemostasis, and immune response, studies in activation of NOD2 in SARS-CoV-2 infection could showed new insight into the pathogenesis and treatment of inflammation and thrombotic complications in COVID-19 disease.

## Platelets immunomodulatory functions

Platelet indicates cellular immunomodulatory functions via having interactions with endothelial cells and leukocytes and responses to infection. Accordingly, these responses may consequently enhance vascular inflammation and induce thrombosis [92, 93]. Previous studies have shown that by considering the presence of TLR-1, − 2, − 4, − 6, and TLR-7 at the membrane and intracellular of platelet, their expressions depend on the status of platelet’s activation [22–24]. TLR-1,-6 in infection situation is responsible for generating pro-inflammatory platelets’ interaction with leukocytes, including neutrophils, monocytes, eosinophils, and dendritic cells that, as innate immune mediated cells, can accelerate platelet’s aggregation [47]. Circulating platelets contain a functional spliceosome, particularly endogenous pre-mRNAs as well as small nuclear ribonucleoproteins [94–96]. During platelet’s activation, splices introns from interleukin 1-β or tissue factor (TF) pre-mRNAs in platelet cytoplasm can be translated into proteins [97, 98]. In this regard, TF overexpression in platelet and monocyte may be related to thrombosis in COVID-19 [99]. During the platelet activation, growth factor cytokines, chemokines, and molecules such as sCD40L, are released. Platelet is the primary source of sCD40L in circulation that plays a critical role in thrombosis and initiating both innate and adaptive immunities [23, 100]. Additionally, platelets express some functional chemokine receptors such as CCR1, 3, 4, and CXCR4, which are involved in infection, hemostasis, inflammation, and even in the development of atherosclerosis. A previously performed study has shown that sCD62P is increases in ARDS patients and in severe and non-survivors compared with non-severe cases and survivors, respectively [37–39]. Therefore, future discoveries related to the immune-mediated activation platelet are necessary to guide the type of therapies needed to control both thrombosis and coagulopathies, particularly in severely ill COVID-19 patients.

## Inhibitors of TLRs and P-selectin

TLRs antagonists include monoclonal antibodies, bacterial-derived proteins, natural or synthetic small molecules. TLR3, TLR7, and TLR8 antagonists can be used against viral infections. Among TLRs, TLR4 is a remarkable pattern recognition receptor recognizes multiple PAMPs of bacteria, viruses, and other pathogens and DAMPs from host lytic cells. Several drugs have been demonstrated to have inhibitory effects on the TLR4 pathways. TLR4 antagonist FP7 significantly decreased the cytokine production in response to lethal lipopolysaccharide (LPS) used in the influenza infection [101]. Eritoran and Tak242 as TLR4 antagonists were developed for the treatment of severe sepsis. TAK-242 (Resatorvid) reduce signaling and inflammation by blocks the interaction between TLR4 and the adaptor proteins TIRAP and TRAM [102]. TAK-242 has pre-clinical success but in clinical investigations the results are not promising. In a phase III trial in managing of severe sepsis, serum cytokine levels suppression of IL-6, IL-8, and TNF-훼 compared to the placebo group have not been shown [103]. Recently, a novel inhibitory activity of angiotensin II receptor blockers (ARBs), and statins on TLR2 and TLR4 signaling was discovered. Valsartan (from the ARB family) has been demonstrated to decrease proinflammatory cytokine release and infarct size by inhibiting TLR4 signaling. Among statins family, Atorvastatin, Fluvastatin, Simvastatin has all shown an inhibitory effect on the reduction in vascular inflammation and the TLR4 signaling and pathway [104–106]. Eritoran (E5564) and TAK-242 are currently undergoing phase III clinical trials, especially for severe sepsis. In septic cases with in the high-dose treatment of eritoran a 12% reduction in the mortality rate compared to placebo was demonstrated [107]. Eritoran blocked DAMP accumulation and attenuated influenza virus-induced acute lung injury [108].

Anti P-selectin monoclonal antibodies or P-selectin antagonist reduces the risk of inflammation and thrombosis [109]. Inclacumab a novel and recombinant monoclonal antibody against P-selectin, block the P-selectin- PSGL-1 mediated cell adhesion. Inclacumab is a human IgG4 monoclonal antibody has anti-cell adhesion effects and the potential of anti-inflammatory, antithrombotic, and antiatherogenic properties [110–112]. Recently crizanlizumab, a human IgG2 anti-P-selectin antibody, approved by FDA. Crizanlizumab reduce vaso-occlusive crises (VOCs) in sickle cell disease patients [113]. These P-selectin antagonists may have benefits to inhibit of platelet-leukocyte-endothelial interactions in COVID-19 patients and reduce the thrombosis complications induced by SARS-COV2 infection.

## Conclusion

Severe SARS-CoV-2 infection mostly presents with coagulation abnormalities, pulmonary microvascular thrombosis, and severe inflammatory response [114]. Thrombosis complications are common among critically ill COVID-19 patients, and these also increase the risk of some life-threatening complications such as myocardial infarction and ARDS [40, 41]. Although the mechanisms of thrombosis in SARS-CoV-2 infection are still unclear, platelet’s activation and inflammatory responses may contribute in this process [42]. TLRs could bind to the specific ligands and then result in the activation of the inflammatory cascades. As well, platelet can be activated by TLRs, and in addition, inflammatory mediators such as LPS and TNF-ɑ may induce P-selectin expression [16, 84]. P-selectin, as a platelet’s activation marker, plays central roles in leukocyte recruitment and expression of TF by monocyte, which is the activator of extrinsic coagulation cascade [43]. Both platelet’s activation and aggregation play critical roles in the pathogenesis of tissue damages such as acute myocardial infarction (AMI) and myocardial ischemia injury [115, 116]. Additionally, local release of DAMPs during the process of AMI is known to trigger proinflammatory TLRs activation, which consequently leads to the aggravation of myocardial injury [19, 20]. It is possible that the alternative platelet activation pathways, which are not targeted by currently available anti-platelet agents, may promote recurrent thrombosis in these settings [117].

SARS-CoV-2 -spike protein attachment to ACE2 on endothelial cells, platelets, and other target cells, triggers the pathogenesis of COVID-19 infection [25, 83]. Previous studies have found that the spike protein could bind to extracellular domains of TLRs, including TLR-1,-4,-6 [25]. Moreover, it has been proposed that the spike protein have a strong affinity with TLR4. Accordingly, TLR4 is mainly present at the surface cell membrane and it recognizes viral proteins before their entrance into the cell and also into the endosomal membrane [118]. TLR4 signaling is important in initiating inflammatory responses, and its overexpression can also lead to hyper inflammation reactions [119–121]. Regarding the fact that platelet could express TLR-4, it may play significant roles in both platelet’s activation and thrombosis. As well, Platelet overexpression of P-selectin in severe SARS-CoV-2 patients suggests a central role of platelet’s activation as a part of the pathogenic mechanism of COVID-19 leading to the production of pulmonary thrombi. So, the administration of anti P-selectin antibody like Crizanlizumab or inclacumab may be helpful for the severe cases. In this regard, a rapid investigation is required to determine the pathways that mostly contribute to platelet’s activation, because these may be important in reducing the rates of morbidity and mortality caused by COVID-19 infection. Among the alternative pathways, TLRs related thrombosis may play a more critical role in COVID-19 complex pathophysiology. In this regard, further studies are required to determine the role of TLRs in the mechanisms of thrombosis and coagulopathies associated with COVID-19 infection. In addition, the administrations of anti-P selectin monoclonal antibody and TLRs antagonist may reduce the cytokine storm, thrombosis, and mortality in COVID-19 patients. More studies are needed to investigate the clinical significance of both TLRs upregulation and antagonist in COVID-19-related thrombosis.

## Data Availability

Not applicable.

## Abbreviations

SARS-CoV-2: severe acute respiratory syndrome coronavirus 2
COVID-19: coronavirus disease
TLRs: toll-like receptors
PAMPs: pathogen-associated molecular patterns
TF: tissue factor
